# Evaluating the impact of stay-at-home orders on the time to reach the peak burden of Covid-19 cases and deaths: does timing matter?

**DOI:** 10.1186/s12889-020-09817-9

**Published:** 2020-11-23

**Authors:** Alexandra Medline, Lamar Hayes, Katia Valdez, Ami Hayashi, Farnoosh Vahedi, Will Capell, Jake Sonnenberg, Zoe Glick, Jeffrey D. Klausner

**Affiliations:** 1 Emory University School of Medicine, Atlanta, Georgia; 2 David Geffen School of Medicine, Los Angeles, California USA; 3 Fielding School of Public Health, Los Angeles, California USA; 4 Stanford University School of Medicine, Stanford, California USA; 5 University of California, Berkeley, California USA

## Abstract

**Background:**

The economic, psychological, and social impact of pandemics and social distancing measures prompt the urgent need to determine the efficacy of non-pharmaceutical interventions (NPIs), especially those considered most stringent such as stay-at-home and self-isolation mandates. This study focuses specifically on the impact of stay-at-home orders, both nationally and internationally, on the control of COVID-19.

**Methods:**

We conducted an observational analysis from April to May 2020 and included both countries and US states with known stay-at-home orders. Our primary exposure was the time between the date of the first reported case of COVID-19 to an implemented stay-at-home mandate for each region. Our primary outcomes were the time from the first reported case to the highest number of daily cases and daily deaths. We conducted linear regression analyses, controlling for the case rate of the outbreak in each respective region.

**Results:**

For countries and US states, a longer period of time between the first reported case and stay-at-home mandates was associated with a longer time to reach both the peak daily case and death counts. The largest effect was among regions classified as the latest 10% to implement a mandate, which in the US, predicted an extra 35.3 days (95% CI: 18.2, 52.5) to the peak number of cases, and 38.3 days (95% CI: 23.6, 53.0) to the peak number of deaths.

**Conclusions:**

Our study supports the association between the timing of stay-at-home orders and the time to peak case and death counts for both countries and US states. Regions in which mandates were implemented late experienced a prolonged duration to reaching both peak daily case and death counts.

## Background

The coronavirus disease 2019 (COVID-19) is an acute respiratory disease spread primarily through the inhalation of infectious droplets and aerosol particles [1, 2]. Since the first case announced on December 8, 2019, in Wuhan, China, COVID-19 has spread internationally with the eventual announcement of a global pandemic by the World Health Organization (WHO) on March 11, 2020 [3]. Healthcare systems and governments worldwide have been under pressure since this designation to implement strategies and containment measures against COVID-19, an unprecedented virus with challenges in all that is left to learn [4].

Extrapolation from epidemiological models of COVID-19 has suggested that intensive physical distancing could “flatten the curve” and prevent the overloading of our health systems [5]. Social distancing measures, aimed at reducing contact between people, include school closings, stay-at-home mandates, and government support for telecommuting [6, 7]. These measures have become commonly adopted practices on a world-wide scale [8], with the goal of reducing the frequency of physical contact and subsequent transmission of the virus between persons [1]. Various degrees of these social distancing measures were employed in the mitigation of previous respiratory viral pandemics such as the Spanish flu pandemic in 1918 and the Severe Acute Respiratory Syndrome (SARS) outbreak in 2003, when clear pharmaceutical treatments or vaccines were unavailable. Although retrospective reviews of these overarching measures suggest overall unestablished impact in quelling the spread of disease [7], the challenges and impracticality of imposing these measures have long been acknowledged [6, 9]. Given the devastating economic, psychological, and social consequences associated with pandemics in general [10] and with COVID-19 specifically [11, 12], there is a need to clearly distinguish between the efficacy of different social distancing measures. In particular, there is a need to evaluate the efficacy of those measures considered most stringent such as stay-at-home and self-isolation mandates.

Pan et al. sought to evaluate the effectiveness of non-pharmaceutical interventions (NPIs) and found that a series of various public health interventions were temporally associated with the improved control of the COVID-19 outbreak in Wuhan, China [13]. Furthermore, their study concluded that the implementation of NPIs was associated with a reduction of the effective reproductive number (R_*t*_), defined as the average number of secondary cases per primary case at calendar time *t* [14], to below 1.0 on February 6, 2020 and to below 0.3 on March 1, 2020 [13]. Since then, many studies aimed at determining the efficacy of social distancing, mostly within the US, have demonstrated the protective effects of NPIs on controlling the spread of COVID-19 [15, 16].

The objective of this study is to add to the growing evidence base on this topic by evaluating the relationship between country and US state stay-at-home orders and the spread of COVID-19 in each region included in our analysis. We quantify the time interval between a country or US state’s first reported case of COVID-19 and its implementation of a stay-at home-order to assess any relationship between stay-at-home orders and their impact both within and outside of the US.

## Methods

### Source of data

We conducted an observational study from April 2020 to May 2020. First, for country-level data, we collected and cross-checked daily case and death counts from WHO daily situation reports [17] and from *worldometer.com* [18]*.* For US states, we used available case and death count data online from each state’s official US Department of Health website as well as *The New York Times* [19] for the date of the implementation of stay-at-home orders. For information related to the date of implementation of social measures on a country level, we referenced government announcements on national or regional official websites or news sites, that were updated daily on regional information related to the pandemic, similar to other studies that have focused on this topic [20]. Google was used as our primary search engine. Specific terms used in our online searches included ‘date of stay-at-home orders 2020,’ ‘non-pharmaceutical interventions COVID-19,’ and ‘stay-at-home mandates.’ We conducted a search for each respective country and US state analyzed in the study.

### Case definitions and outcome measures

Stay-at-home orders were defined as regionwide restrictions of non-essential internal movement (commonly referred to as “lockdowns”) [21]. Inclusion criteria for our study were states or countries that imposed region-wide stay-at-home orders with publicly available dates of implementation. Regions that implemented other social distancing strategies, such as “curfews”, but not stay-at-home orders, were not included in our analysis. Similarly, for the purpose of maintaining the integrity of comparing region-wide mandates, regions that did not implement country-wide stay-at-home orders were also excluded. To assess the association between stay-at-home orders and their impact, we measured the number of days between the implementation of a regional stay-at-home order and objective measures of the peak COVID-19 burden for each US state and country. We chose two main outcome variables to reflect this peak, which included: 1. Highest daily case count, 2. Highest daily death count. The highest daily case count was defined as the largest number of laboratory-confirmed cases and the highest daily death count as the largest number of new deaths attributed to COVID-19 per day.

Our primary exposure was the number of days between the first reported case of COVID-19 in a studied area and the date of nation- or state-wide restriction of internal movement. We chose to measure the peak from the date of the first reported case of COVID-19 in each region to account for the variation in the timing of the pandemic across both the globe and the US. This variable was measured as both a continuous and categorical variable. Each location, based on the number of days between its first case and its stay-at-home mandate, was categorized into one of three equal terciles: early, middle, or late, analyzed with the creation of dummy variables. In addition, based on the frequency distribution for both countries and US states, the earliest and latest 10% to implement mandates were also formed into their own categories. Our primary outcome variables were the number of days from the first reported case of COVID-19 to the peak of daily cases and to the peak of daily deaths, in each respective country and US state included in our analysis.

### Data analysis

We conducted linear regression analyses, controlling for the regional case rate of the outbreak which was defined as the number of new cases per 100,000 persons on the day that the mandate was implemented. The analysis was conducted for countries and US states. We used SPSS® Version 26 for our analysis with a significance level of .05.

## Results

### US state-level descriptive analysis

**Fig. 1:**
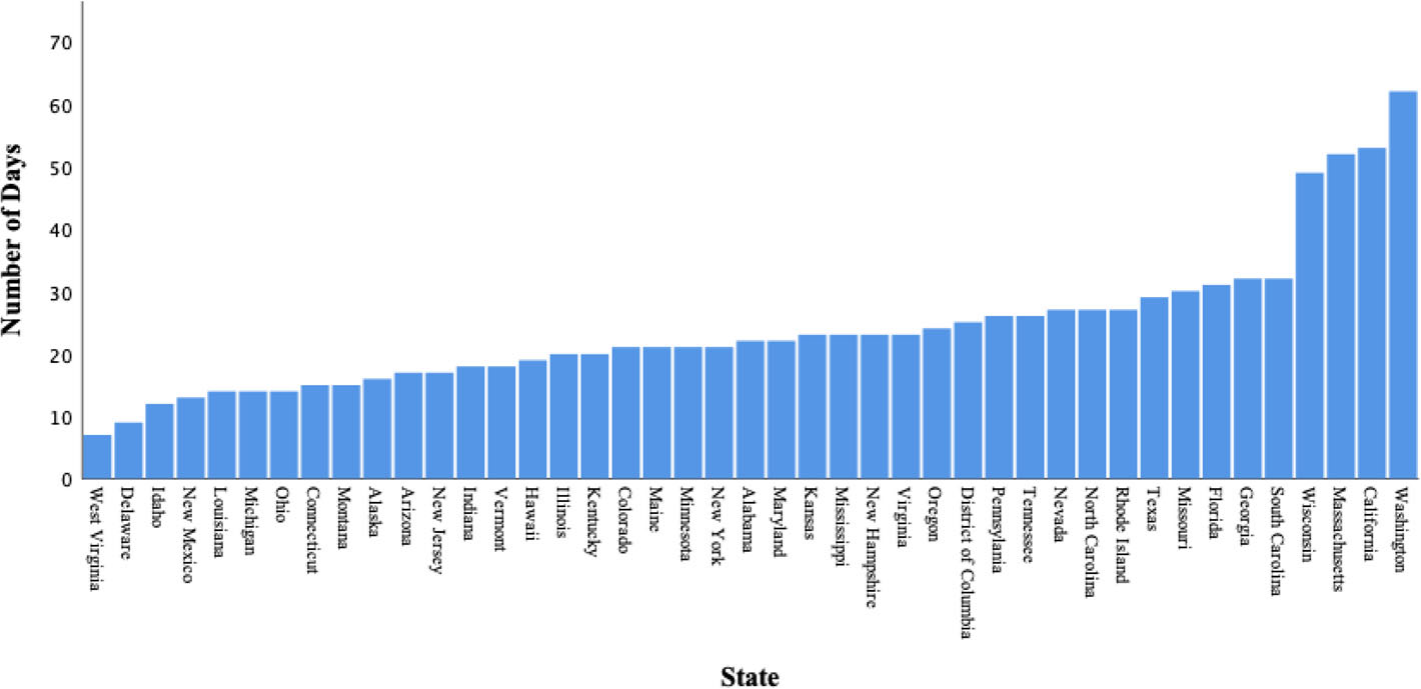
Number of Days Between Date of First Reported Case and Stay-at-Home Mandate per US State (*n* = 43)

**Fig. 2:**
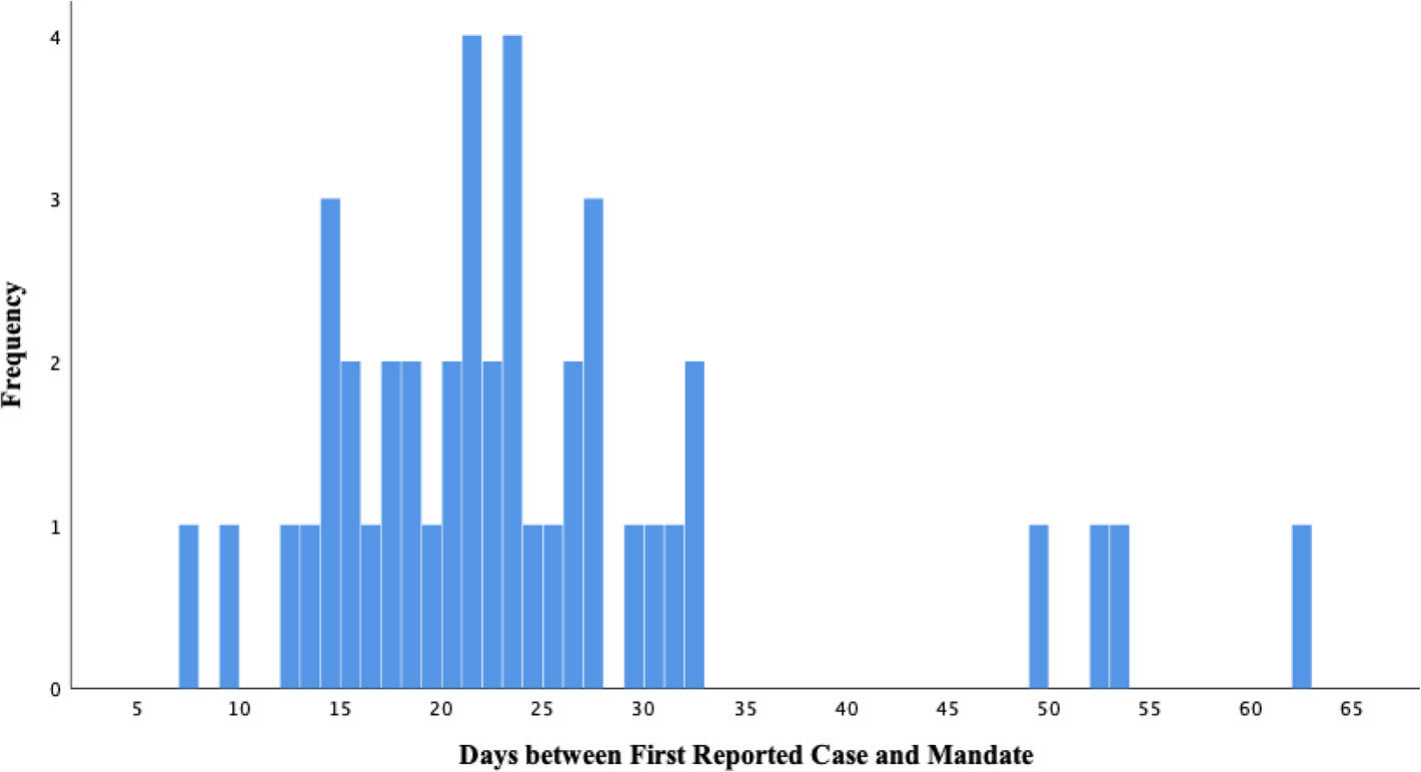
Distribution of the Number of Days Between Date of First Reported Case and Stay-at-Home Mandate per US State (n = 43)

Forty-three states with stay-at-home orders were included in our analysis. Of the 43 states included, the number of days between the first reported case and the stay-at-home mandate ranged from 7 to 62 days (Fig. 1), with a mean of 24.0 days and a standard deviation of 11.5 days (Fig. 2).

### Country-level descriptive analysis

**Fig. 3:**
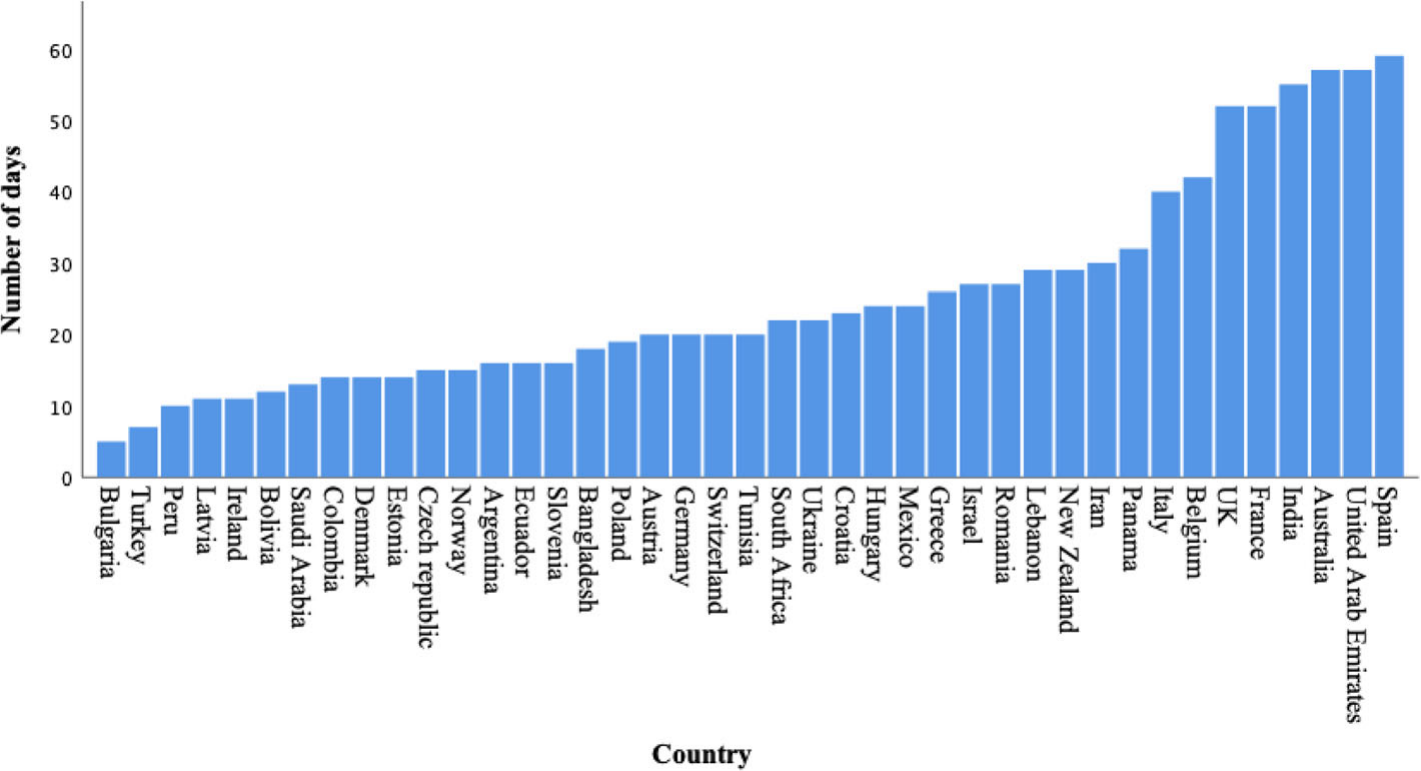
Number of Days Between Date of First Reported Case and Stay-at-Home Mandate per Country (*n* = 41)

**Fig. 4:**
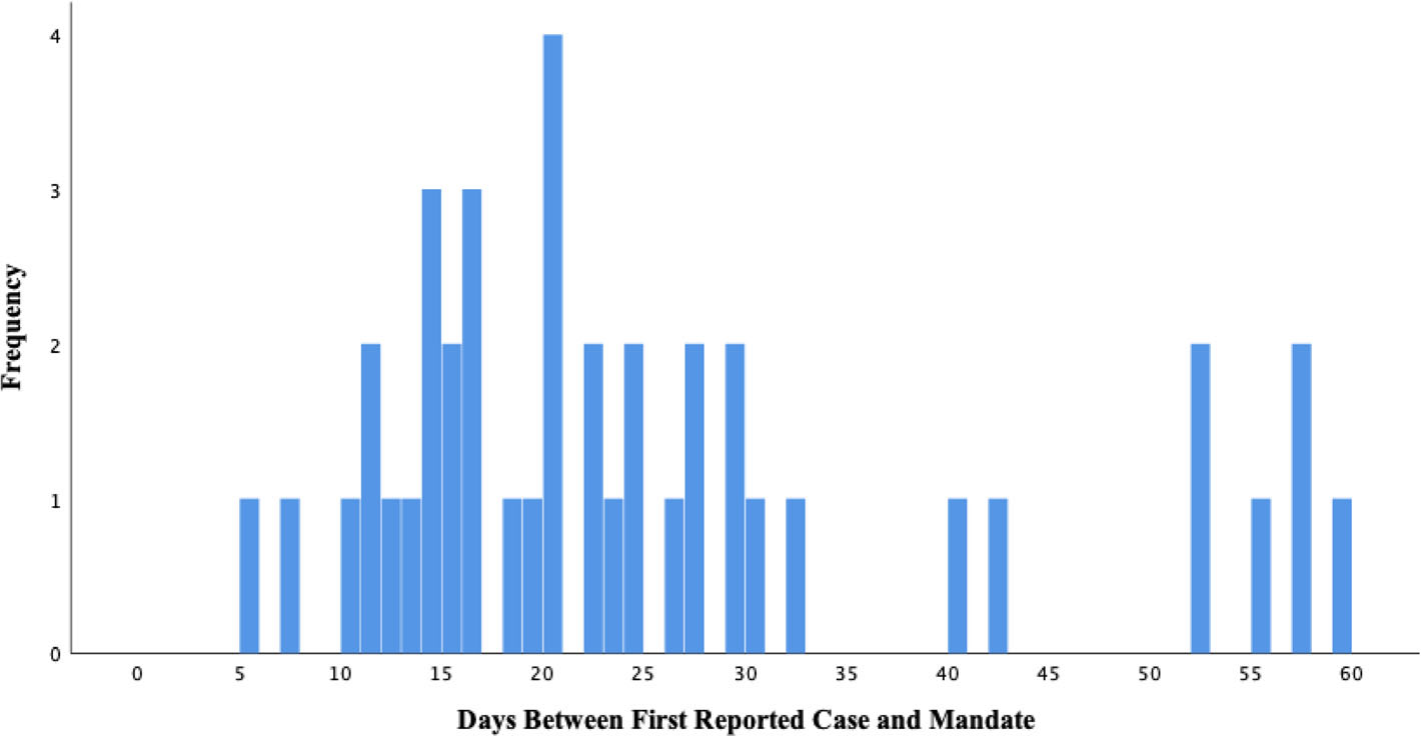
Distribution of the Number of Days Between Date of First Reported Case and Stay-at-Home Mandate per Country (n = 41)

Forty-one countries with stay-at-home orders were included in our analysis. Of the 41 countries included, the number of days between the first reported case and the stay-at-home mandate ranged from 5 to 59 days (Fig. 3), with a mean of 25.2 days and a standard deviation of 14.9 days (Fig. 4).

### Linear regression analysis

A total of 12 linear regression models were conducted to analyze the effect of the timing of stay-at-home mandates, represented both as a continuous and categorical variable, on daily case and death rates. For both country and US state-level-data, a larger number of days between the first reported case and the stay-at-home mandate was associated with a longer time to reach both the peak of daily cases and deaths for each respective region, as represented by the beta coefficients for each of the 12 respective linear regression models (Tables 1 and 2). For US states, each additional day added between the first reported case and the implementation of a mandate predicted an extra 1.1 days to reach the peak number of cases (95% CI: 0.7, 1.5) and an extra 1.0 days to reach the peak number of deaths (95% CI: 0.7, 1.4). The largest effect was among regions classified as the latest 10% to implement a mandate, which in the US, predicted an extra 35.3 days (95% CI: 18.2, 52.5) to the peak number of cases, and 38.3 days (95% CI: 23.6, 53.0) to the peak number of deaths. No significant effect was seen for the countries and states that were identified as the earliest 10% of regions to implement their mandates, respectively. Classifying states and countries into categorical terciles yielded mixed results, elucidating stronger associations for state-level compared to country-level data.

**Table 1:** Linear Regression Models Predicting Number of Days to Highest Case and Death Count for State-level Analysis (n = 43)

*Method of Classifying Exposure Variable (Number of Days Between 1st Reported Case and Mandate)*	Measured Effect on Peak A: Number of Days from First Reported Case to Highest Number of Daily New Deaths **		
	Coefficient	95% CI	***P***-value
Continuous Variable	1.1	.65, 1.5	.000*
Categorical Terciles: Early, middle, late	13.1	6.9, 19.3	.000*
*Early* vs. *middle/late*	−24.1	−34.5, −13.8	.000*
*Middle* vs. *early/late*	8.5	−3.8, 20.8	*.17*
*Late* vs. *early/middle*	14.8	2.9, 26.6	***.016***
Categorical: *Earliest 10%*	−18.5	−38.4, 1.3	*.067*
Categorical: *Latest 10%*	35.3	18.1, 52.5	.000*
	Measured Effect on Peak B: Number of Days from First Reported Case to Highest Number of Daily New Deaths **		
	**Coefficient**	**95% CI**	***P*-value**
Continuous Variable	1.0	0.7, 1.4	.000*
Categorical Terciles: Early, middle, late	10.7	4.7, 16.8	.001*
*Early* vs. *middle/late*	−15.5	−26.4, −4.2	.007*
*Middle* vs. *early/late*	−1.2	−12.9, 10.5	*.843*
*Late* vs. *early/middle*	16.3	5.6, 26.9	***.004***
Categorical: *Earliest 10%*	−11.3	−30.2, 7.6	*.234*
Categorical: *Latest 10%*	38.3	23.6, 53.0	.000*

*Significant results at *p* < 0.05

**Models controlled for case rates per region, defined as number of new daily cases per 100,000 persons on the date of the implemented mandate

**Table 2:** Linear Regression Models Predicting Number of Days to Highest Case and Death Count for Country-level Analysis (n = 41)

*Method of Classifying Exposure Variable (Number of Days Between 1st Reported Case and Mandate)*	Measured Effect on Peak A: Number of Days from First Reported Case to Highest Number of Daily New Cases **		
	Coefficient	95% CI	***P***-value
Continuous Variable	0.7	0.2, 1.1	.000*
Categorical Terciles: Early, middle, late	10.2	1.6, 18.8	.021*
*Early* vs. *middle/late*	−13.1	−28.5, 2.3	*.093*
*Middle* vs. *early/late*	−4.2	−19.9, 11.5	*.592*
*Late* vs. *early/middle*	17.4	2.5, 32.3	.023*
Categorical: *Earliest 10%*	−7.6	−32.8, 17.5	*.543*
Categorical: *Latest 10%*	30.0	6.9, 53.2	.012*
	Measured Effect on Peak B: Number of Days from First Reported Case to Highest Number of Daily New Deaths **		
	**Coefficient**	**95% CI**	***P*-value**
Continuous Variable	.5	0.2, 0.9	.002*
Categorical Terciles: Early, middle, late	6.1	−0.5, 12.6	*.068*
Early vs. middle/late	−7.4	−18.9, 4.1	*.201*
Middle vs. early/late	−3.2	−14.8, 8.4	*.582*
Late vs. early/middle	10.6	−0.6, 21.9	*.063*
Categorical: *Earliest 10%*	−4.7	−23.3, 8.5	*.609*
Categorical: *Latest 10%*	26.3	9.9, 42.7	.002*

*Significant results at *p* < 0.05

**Models controlled for case rates per region, defined as number of new daily cases per 100,000 persons on the date of the implemented mandate

## Discussion

Our study builds on emerging epidemiological data supporting the efficacy of NPIs, and specific to our study, stay-at-home mandates, in the control of the COVID-19 pandemic [8, 21–28]. Recent epidemiologic studies have shown that the COVID-19 pandemic can be suppressed by a lockdown [29], however, novel to our study is the elucidation of the importance of the timing of the implementation of these measures. Notably, when the timing of mandate implementation was analyzed as a continuous variable, the effect on timing to peak case and death counts was modest with an increase in the time to peak of approximately one day. This mild effect could reflect variation between regions. Thu et al. reported similar findings on the effects of social distancing measures in ten highly infected countries. These investigators found that there was great variation in the effectiveness of social distancing measures between the countries included in their analysis [20]. By contrast, in our study, a relatively strong effect was demonstrated for regions categorized categorically as late mandate implementers, with these regions corresponding to the largest predicted prolongation in the number of days to peak daily case and death counts. This strong association supports the possibility of a “threshold” date or range of dates only until which an implemented mandate may be efficacious.

Contrary to our findings, a recent deterministic compartmental transmission modeling study found that short-term government-imposed social distancing alone would delay but not reduce the peak number of COVID-19 diagnoses [30]. These authors proposed that more timely imposed social distancing may be beneficial by allowing time for healthcare systems and public health regional leaders to prepare for an increasing burden of cases [30]. Conversely, one may argue that earlier peaks may instead be preferrable from a public health perspective, since overall case counts may be subsequently lower. If one considers two theoretical epidemiologic curves with the same peak number of cases but with one reaching its peak earlier than the other, the earlier curve given all else equal would have a smaller total case count given a smaller area underneath its curve.

Strengths of this study include the temporality of the interventions and outcomes included in our analysis, which supports biological plausibility. Furthermore, our study included multiple iterations of analyses to support the observed trend. Our findings were replicated both for US states as well as for our included countries, which supports the consistency of the observed effect. Finally, we accounted for the relative burden of disease at the time of each region’s mandate, by controlling for the case rate of disease for each country and US state included in our regression models.

The main limitation of this study was its observational nature and the exclusion of other NPIs, possibly confounding, that were implemented in the various regions we analyzed. However, we assume that by virtue of including many different regions and by repeating our analysis in several different ways, it can be assumed that the overall preventative effect of these NPIs were evenly spread out across these regions [27]. Furthermore, another limitation of our study is that our analysis did not account for the fidelity of and adherence to the implemented mandates which may have therefore biased our results. However, the directionality of this bias is unknown. Another limitation is the two-month duration of our study which did not capture secondary peaks within states and countries and varying termination processes of different regions [20]. However, for all US and for many countries included in our study, the peak incidence of case and death counts had already been reached far before the end date of our epidemiologic observation. Finally, the differences between regions as well as changes in testing capacity within each respective region may have also largely impacted the results of this study, as alluded to in other epidemiological observational studies that have recently investigated this topic [8, 23].

## Conclusions

Overall, our study supports the association between the timing of stay-at-home mandates and the peak number of cases and deaths of COVID-19. This association demonstrates the potential beneficial effect of earlier stay-at-home mandates in the control of the spread of this pandemic. Earlier stay-at-home mandates corresponded with earlier peaks and theoretically smaller overall regional burdens of infection. While the observed effect was generally modest, regions that significantly delayed implementation of their stay-at-home mandates experienced a pronounced and prolonged delay in reaching both peak daily case and death counts of COVID-19. This has important implications for policy leaders moving forward in the control of COVID-19 and other potential future pandemics, to consider implementing regional stay-at-home mandates as preventative rather than responsive measures.

## Data Availability

The datasets during and/or analyzed during the current study available from the corresponding author on reasonable request.

## Abbreviations

NPIs: Non-pharmaceutical interventions
COVID-19: Coronavirus disease 2019
WHO: World Health Organization
SARS: Severe Acute Respiratory Syndrome
R_*t*_: Effective reproductive number

## References

[1] Singhal T (2020). A review of coronavirus Disease-2019 (COVID-19). Indian J Pediatr.

[2] Lotfi M, Hamblin MR, Rezaei N (2020). COVID-19: transmission, prevention, and potential therapeutic opportunities. Clin Chim Acta.

[3] Guo YR, Cao QD, Hong ZS (2020). The origin, transmission and clinical therapies on coronavirus disease 2019 (COVID-19) outbreak - an update on the status. Mil Med Res.

[4] Kassem AM (2020). COVID-19: mitigation or suppression?. Arab J Gastroenterol.

[5] Gostin LO, Wiley LF. Governmental Public Health Powers During the COVID-19 Pandemic: Stay-at-home Orders, Business Closures, and Travel Restrictions. JAMA. 2020;323(21):2137–8. 10.1001/jama.2020.5460. 10.1001/jama.2020.5460 32239184

[6] Inglesby TV, Nuzzo JB, O'Toole T, Henderson DA (2006). Disease mitigation measures in the control of pandemic influenza. Biosecur Bioterror.

[7] Adalja AA, Toner E, Inglesby TV (2020). Priorities for the US health community responding to COVID-19. JAMA..

[8] Eubank S, Eckstrand I, Lewis B, Venkatramanan S, Marathe M, Barrett CL (2020). Commentary on Ferguson, et al., "impact of non-pharmaceutical interventions (NPIs) to reduce COVID-19 mortality and healthcare demand". Bull Math Biol.

[9] Bell D, Nicoll A, Fukuda K (2006). Non-pharmaceutical interventions for pandemic influenza, national and community measures. Emerg Infect Dis.

[10] Barrett C, Bisset K, Leidig J, Marathe A, Marathe M (2011). Economic and social impact of influenza mitigation strategies by demographic class. Epidemics..

[11] Açikgöz Ö, Günay A (2020). The early impact of the Covid-19 pandemic on the global and Turkish economy. Turk J Med Sci.

[12] Nicola M, Alsafi Z, Sohrabi C (2020). The Socio-Economic Implications of the Coronavirus and COVID-19 Pandemic: A Review. Int J Surg.

[13] Pan A, Liu L, Wang C, et al. Association of Public Health Interventions With the Epidemiology of the COVID-19 Outbreak in Wuhan, China. JAMA. 2020;323(19):1915–23. 10.1001/jama.2020.6130. 10.1001/jama.2020.6130 PMC7149375 32275295

[14] Nishiura H, Chowell G. The Effective Reproduction Number as a Prelude to Statistical Estimation of Time-Dependent Epidemic Trends. Math and Stat Estimation Approaches Epidemiol. 2009;103–21. 10.1007/978-90-481-2313-1_5.

[15] Courtemanche C, Garuccio J, Le A, Pinkston J, Yelowitz A. Strong Social Distancing Measures In The United States Reduced The COVID-19 Growth Rate. Health Aff (Millwood). 2020;39(7):1237-46. . 10.1377/hlthaff.2020.00608 32407171

[16] Lin G, Zhang T, Zhang Y, Wang Q. Statewide Stay‐at‐Home Directives on the Spread of COVID‐19 in Metropolitan and Nonmetropolitan Counties in the United States. J Rural Health. 2020. 10.1111/jrh.12464. 10.1111/jrh.12464 PMC7272888 32391620

[17] Organization WH. Coronavirus disease (COVID-19) weekly epidemiological update and weekly operational update. https://www.who.int/emergencies/diseases/novel-coronavirus-2019/situation-reports. Published 2020. Accessed.

[18] https://www.worldometers.info/coronavirus/. Published 2020. Accessed.

[19] Mervosh SL, Denise Lu; swales, Vanessa see which states and cities have told residents to stay at home. The New York times https://www.nytimes.com/interactive/2020/us/coronavirus-stay-at-home-order.html. Published 2020. Accessed.

[20] Thu TPB, Ngoc PNH, Hai NM, Tuan LA (2020). Effect of the social distancing measures on the spread of COVID-19 in 10 highly infected countries. Sci Total Environ.

[21] Siedner MJ, Harling G, Reynolds Z, Gilbert RF, Venkataramani A, Tsai AC. Social distancing to slow the U.S. COVID-19 epidemic: an interrupted time-series analysis. medRxiv. 2020; 2020.2004.2003.20052373.

[22] Thunström L, Newbold S, Finnoff D, Ashworth M, Shogren J. The Benefits and Costs of Using Social Distancing to Flatten the Curve for COVID-19. J Benefit-Cost Analysis. 2020;11(2):179-95. 10.1017/bca.2020.12.

[23] Dreher N, Spiera Z, McAuley FM, et al. Impact of policy interventions and social distancing on SARS-CoV-2 transmission in the United States. medRxiv. 2020; 2020.2005.2001.20088179.

[24] Dehning J, Zierenberg J, Spitzner FP, et al. Inferring change points in the spread of COVID-19 reveals the effectiveness of interventions. Science. 2020;369(6500):eabb9789. 10.1126/science.abb9789 PMC7239331 32414780

[25] Strong Social Distancing Measures In The United States Reduced The COVID-19 Growth Rate. *Health Affairs.*0(0):10.1377/hlthaff.2020.00608. 10.1377/hlthaff.2020.00608 32407171

[26] Wagner AB, Hill EL, Ryan SE, et al. Social Distancing Has Merely Stabilized COVID-19 in the US. medRxiv. 2020; 2020.2004.2027.20081836.

[27] Banholzer N, van Weenen E, Kratzwald B, et al. Impact of non-pharmaceutical interventions on documented cases of COVID-19. medRxiv. 2020; 2020.2004.2016.20062141.

[28] Nussbaumer-Streit B, Mayr V, Dobrescu AI (2020). Quarantine alone or in combination with other public health measures to control COVID-19: a rapid review. Cochrane Database Syst Rev.

[29] Atalan A (2020). Is the lockdown important to prevent the COVID-9 pandemic? Effects on psychology, environment and economy-perspective. Ann Med Surg (Lond).

[30] Teslya A, Pham TM, Godijk NG, Kretzschmar ME, Bootsma MCJ, Rozhnova G (2020). Impact of self-imposed prevention measures and short-term government-imposed social distancing on mitigating and delaying a COVID-19 epidemic: a modelling study. PLoS Med.

